# Microgeographic variations in Burkitt's lymphoma incidence correlate with differences in malnutrition, malaria and Epstein–Barr virus

**DOI:** 10.1038/sj.bjc.6605947

**Published:** 2010-11-23

**Authors:** P O Sumba, E W Kabiru, E Namuyenga, N Fiore, R O Otieno, A M Moormann, A S Orago, P F Rosenbaum, R Rochford

**Affiliations:** 1 Department of Global Health, Kenya Medical Research Institute, P.O. Box 1578, Kisumu, Kenya; 2 Department of Pathology, Kenyatta University, P.O. Box 43844-00100 Nairobi, Kenya; 3 Department of Zoology, Maseno University, Private Bag-40105, Maseno University, Maseno, Kenya; 4 Department of Microbiology and Immunology, 750 East Adams Street, SUNY Upstate Medical University, Syracuse, NY 13066, USA; 5 Department of Pediatrics, University of Massachusetts Medical School – Biotech II, 373 Plantation Street, Suite 318, Worcester, MA 01605, USA; 6 Kenya National AIDS Control Council, Argwings Kodhek Road, P.O. Box 61307, 00200 Nairobi, Kenya

**Keywords:** selenium deficiency, Epstein–Barr virus (EBV), malaria, endemic Burkitt's lymphoma, pediatric cancer, malnutrition

## Abstract

**Background:**

Endemic Burkitt's lymphoma (eBL) has been associated with Epstein–Barr virus (EBV) and holoendemic *Plasmodium falciparum* malaria. But recent evidence suggests that other risk factors are involved.

**Methods:**

We hypothesised that selenoprotein glutathione peroxidase (GPx), a surrogate of nutritional status, is an important biomarker for eBL risk. We measured plasma GPx, anthropometric markers of malnutrition, EBV viral loads and malaria parasitaemia in children aged 1–9 years (*n*=258) from two locations in Nyanza Province, Kenya, with higher-than-expected and lower-than-expected incidence of eBL. The study participants were malaria asymptomatic children from the community.

**Results:**

Children from eBL high-incidence areas had significantly lower GPx levels, high EBV viral load and more evidence of chronic malnutrition than children from eBL low-incidence areas (all *P*<0.001). Additionally, GPx levels were significantly lower in children with the highest EBV viral load and for those with *P. falciparum* infections (*P*=0.035 and *P*=0.004, respectively).

**Conclusions:**

These results suggest that selenium deficiency may be a risk factor for eBL.

Endemic Burkitt's lymphoma (eBL) is the most prevalent paediatric cancer in sub-Saharan Africa (Parkin *et al*, 2008). The aetiology of this cancer is thought to be multifactorial, with malaria and Epstein–Barr virus (EBV) infections being two well-documented co-factors (reviewed in Rochford *et al*, 2005; Kelly and Rickinson, 2007), although it is likely that there are other co-factors. One area that has had little attention in studies on the aetiology of eBL is the potential role of micronutrient deficiency and, in particular, selenium deficiency. Selenium is an essential micronutrient and is an integral component of the antioxidant enzyme glutathione peroxidase (GPx) (Rotruck *et al*, 1973). Low GPx activity has been correlated with enhanced risk of cancer in certain populations (Zhuo *et al*, 2009). However, there are little data on selenium and GPx levels in populations at risk for eBL.

Nyanza Province in western Kenya is an area with a high burden of both eBL and malaria (Rainey *et al*, 2007a). We had previously found a strong correlation between endemic malaria and eBL incidence (Rainey *et al*, 2007a), consistent with the earliest studies of eBL (Wright, 1967; Morrow, 1985). However, when we performed a more detailed examination of eBL incidence in Nyanza Province, we found microgeographic variation of eBL cases with closely spaced clusters that had higher-than-expected or lower-than-expected incidence of eBL (Rainey *et al*, 2007b). These variations in eBL incidence led us to hypothesise that other environmental factors were affecting the risk for eBL. In the current study, we took advantage of these two closely situated regions to perform a cross-sectional study investigating whether differences in plasma GPx levels in children existed between these two regions and whether GPx levels correlated with peripheral EBV viral loads and/or malaria burden.

## Materials and methods

Kenya is divided administratively into five administrative levels: provinces, districts, divisions, locations and sub-locations. Sub-locations consist of villages and towns. The study involved healthy children (*n*=258) who were permanent residents of a sub-location that experienced higher-than-expected cases of eBL (e.g., Kanyawegi) and sub-locations with lower-than-expected cases of eBL (e.g., North and South Kowe) (Rainey *et al*, 2007b). Kanyawegi is in Nyanza Province, Kisumu East District, Winam Division, SW Kisumu location. North and South Kowe are in North Seme Location, Kombewa Division, Kisumu West District. Between 1999 and 2004, the average annual incidence of eBL in Kowe sub-locations was 0.00 eBL cases per 10 000 children, whereas in Kanyawegi it was 1.32 eBL cases per 10 000 children (Rainey *et al*, 2007b). These two sites are referred to herein as the low eBL incidence region (Kowe) and the high eBL incidence region (Kanyawegi). Malaria transmission in Kisumu District was identified as Lake Endemic (Snow *et al*, 1998), which is defined as areas where transmission occurs year-round with parasitaemia >50% among the childhood population. Immunity is acquired before adulthood and children and pregnant women have highest disease risk.

Study participants aged between 1 and 9 years were randomly recruited within the two adjacent regions with divergent eBL incidence. The study began by community mobilisation and performing a demographic survey. For ease of household identification, collection of ecological information and generation of required study numbers after preliminary computation of the demographic data, GIS mapping was done using a hand-held GPS satellite-empowered Dell PDA. Information collected was downloaded onto a computer supported with GeoExplorer CE module (Trimble, Inc., Westminister, CO, USA). Study participants were enroled using written informed consent forms through home visits or at organised centres within the study sites. The approved ethical form detailing the purpose of the study and contact persons was availed and duly signed after conducting study participants or their guardians through the reading of the form using a language of choice. Inclusion criteria were residence in the study areas for the past 6 months, absence of severe illness and consenting to the study. The exclusion criteria were presence of severe chronic anaemia (haemoglobin <5 g per 100 ml), blood transfusions within the last 6 months, fever >37.5 °C and residence in an orphanage. The study was approved by the institutional review boards of the SUNY Upstate University and the ethical review committee for the KEMRI (Kenya Medical Research Institute).

Data collection included a questionnaire, completed using face-to-face interviews with guardians as well as a venipuncture blood sample, auxilliary temperature (°C) and height and weight measurements of study participants. Non-fasting blood was collected in the field between 2006 and 2008. Variations were minimised by using the same types of blood collection and processing tubes. Between 5 and 9 ml of blood was collected by venipuncture into heparinised tubes. Plasma was separated by centrifugation under subdued light in a Biosafety hood in the KEMRI laboratory. The samples were then frozen at −80 °C in freezers. Haemoglobin measurements (g per 100 ml) were conducted on site with a portable Hemocue (USA) before confirmation by Beckman Coulter AcT diff2 (Beckman-Coulter Corporation, Miami, FL, USA) in the laboratory. Finger-prick blood samples were collected in EDTA tubes and stored at −80 °C for later DNA extraction. Thick and thin blood smears were prepared for malaria parasite screening and density calculation following the WHO criteria. The number of asexual *Plasmodium* parasites per 200 leukocytes was determined and parasite density was calculated by multiplying with the actual white blood cell count.

Levels of GPx were determined in plasma samples by enzyme-linked immunosorbent assays (ELISAs) using a commercially available kit (BIOXYTECH Plasma pGPx Enzyme Immunoassays, Oxis Research, Portland, OR, USA) and following the manufacturer's protocol. The ELISA plate was read through a computer-aided ELISA reader. Results were interpolated from standard curves generated with recombinant human GPx-selenium protein supplied by the manufacturer. GPx levels (in *μ*g per 100 ml) were determined in a subset of participants, *n*=65 and *n*=93, from the low and high eBL incidence regions, respectively.

DNA was extracted from 200 *μ*l whole blood using Qiagen DNAeasy kit (Qiagen, Valencia, CA, USA) according to the manufacturer's protocol. DNA was eluted off the column in an equivalent volume of H_2_O and stored at −20 °C. DNA (5 *μ*l) was used to determine EBV viral loads by real-time Q-PCR and the viral loads were calculated as viral copies per *μ*g total DNA as previously described (Moormann *et al*, 2005). Viral loads were evaluated in a subset of study participants, *n*=104 from the low eBL IR area *vs n*=127 from the high eBL IR area.

Weight and height measurements were taken for use in the determination of whether the children were wasted, stunted or underweight, based on *Z*-scores <−2. Height-for-age (stunting), weight-for-height (wasting) and weight-for-age (underweight) *Z*-scores were calculated using growth reference curves developed by the NCHS (National Center for Health Statistics) using Epi Info 6.04 (CDC, Atlanta, GA, USA).

### Statistical analyses

The statistical analyses were performed using SPSS (now called PASW; Chicago, IL, USA); for windows (versions 17.0 and 18.0) (IBM CO.) and GraphPad Prism for windows software (version 5.01) (GraphPad Software Inc., San Diego, CA, USA). Means were compared using the *t*-test and one-way analysis of variance (ANOVA), medians were compared with the Mann–Whitney *U*, whereas proportions were evaluated using Pearson's *χ*^2^ test. Statistical associations between variables were further examined using Pearson's correlation coefficient. Variables with significant skewness were transformed towards normality. The *P-*values of <0.05 were considered statistically significant for all analyses. Multivariable analyses were also conducted using logistic regression to evaluate associations between possible cofactors (e.g., malaria and EBV) and locale (e.g., low or high eBL regions) with control for child age. Variables evaluated in this model included continuous GPx (*μ*g per 100 ml), malaria parasite positivity (no, yes), log-transformed EBV levels and child age in years. GPx was also categorised into quartiles to investigate a possible nonlinear association between GPx levels and locale. Logistic regression models included odds ratios (ORs) and 95% confidence intervals (CIs).

## Results

We took advantage of disparate incidence rates of eBL in proximally located villages in Nyanza Province Kenya to ask whether there were other biological factors that would affect eBL incidence rates. We recruited children between the ages of 1 and 9 years with an equal distribution of children between children aged 1–4 and 5–9 years (Table 1). We chose these age ranges as children <5 years have the greatest risk for severe malaria disease, whereas children between 5 and 9 years have the highest incidence of eBL (Rainey *et al*, 2007b). The demographic and clinical characteristics of the study participants (*n*=258) are presented in Table 1. Mean ages were similar in both groups, 5.08 s.d. 2.57) and 4.96 (s.d. 2.38) years, for the low and high eBL incidence regions, respectively. Of the participants, 46% were male, with no difference in gender distribution noted between the two sites (*P*=0.68). Children residing in a high eBL incidence region were found to have lower haemoglobin concentration compared with those from the eBL low-risk area (*P*=0.016) and more cases of anaemia (<11 g per 100 ml, 1–4 years of age; or <11.5 g per 100 ml, 5–9 years of age) (*P*<0.001) compared with children from a low eBL incidence area.

**Figure 1:**
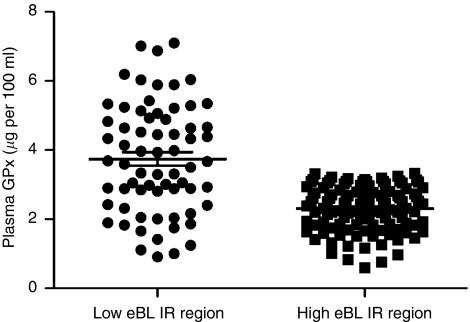
Plasma levels of glutathione peroxidase enzyme (GPx) from children residing in regions with low and high eBL incidence. Plasma levels of GPx in individuals from eBL low-incidence (*n*=65) and eBL high-incidence (*n*=93) regions were measured by enzyme-linked immunosorbent assay (ELISA). The dots represent individual GPx selenium levels and the line through the dots represents the mean level. Differences between groups were statistically significant by *t*-test (*P*<0.0001).

To determine if there were differences in the levels of selenoprotein GPx, we measured GPx in plasma by ELISA in a subset of children from eBL low-incidence (*n*=65) and high-incidence areas (*n*=93). The demographic and clinical characteristics of study participants analysed for GPx levels are shown in Table 2. Children in this subset are representative of the larger study population, as indicated by comparable percentages and means and distribution of parameters measured. Mean plasma selenium levels were significantly higher among the children from the eBL low-incidence region compared with those from the eBL high-incidence region; means of 3.76 (1.58) and 2.36 (0.65) *μ*g per 100 ml, respectively, were observed (*P*<0.001; Table 3 and Figure 1). GPx levels did not vary significantly by age group or gender.

Because GPx is a selenium-based enzyme, we wanted to further examine these children for evidence of malnutrition, as decreased GPx could be an indicator of underlying malnutrition. We evaluated the *Z*-scores between the two populations and found the results presented in Table 4. Comparison of the nutritional status between children from eBL low- and high-risk regions showed that the proportion of children with any indicator of the malnutrition (e.g., stunting, wasting or underweight) was greater among those residing in eBL high-risk region than the ones residing in eBL low-risk regions (*P*=0.043). When comparisons between sites were made analysing unique indicators of malnutrition, we observed a significantly higher proportion of children with stunting from the eBL high-risk area than the eBL low-risk area (*P*=0.003). The proportion of children who were wasted or underweight was comparable between eBL low- and high-risk regions (*P*=0.467 and *P*=0.390, respectively). Further analyses showed that children aged 1–4 years had a greater proportion of individuals with wasting condition than those aged 5–9 years (*P*=0.006). The rest of the malnutrition conditions did not differ significantly between the two age groups. The proportion of children who were wasted, stunted, underweight or had any of the malnutrition conditions did not differ significantly based on gender.

**Figure 2:**
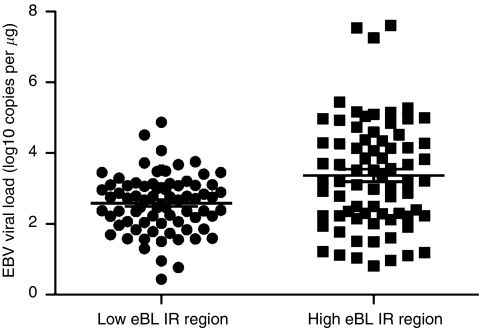
EBV viral load in children residing in regions with low and high eBL incidence. EBV viral load in individuals from eBL low-risk and eBL high-risk region were determined by Q-PCR. The dots represent individual viral loads, whereas the line through the dots represents the mean. Only those study participants with a positive viral load were analysed (*n*=74, eBL low risk and *n*=59, eBL high risk) and are shown. Differences between groups were statistically significant by *t*-test (*P*<0.0001).

In areas where malaria transmission is perennial and intense, EBV persistence is altered, resulting in elevated EBV viral loads in young children compared with children with minimal exposure to malaria (Moormann *et al*, 2005). In addition, children have elevated viral loads during an episode of acute malaria (Rasti *et al*, 2005; Donati *et al*, 2006) that decline following treatment for malaria (Donati *et al*, 2006). The high viral load as a consequence of malaria infection is thought to underlay the mechanisms that increase the risk for the emergence of a malignant clone in children living in malaria endemic regions (Rochford *et al*, 2005). To evaluate whether there were differences in the mean EBV viral loads between the two study sites, DNA was extracted from whole blood and Q-PCR was done to determine EBV viral load in children from eBL low-incidence (*n*=104) and high-incidence regions (*n*=127) (Figure 2). Detectable EBV viral levels were observed in 71% of participants from the low eBL region compared with 46% from the high eBL study areas (*P*<0.001). Among those with detectable EBV, the log viral loads were significantly higher in children residing in the region with high eBL incidence (*n*=59) compared with the low eBL incidence area (*n*=74); the log viral load means were 3.33 (s.d. 1.56) and 2.57 (s.d. 0.81), respectively (*P*<0.001).

We determined the presence of asymptomatic parasitaemia in the study participants. We found that the prevalence of individuals with malaria parasitaemia was significantly different between study sites (*P*<0.001), with 62% of children from high eBL incidence areas positive for malaria parasites compared with 22% from the low eBL area. The mean temperature of children between both sites was within the normal range (36.4 *vs* 36.6°C), indicating that the children included in the study were experiencing asymptomatic parasitaemia. Of the children who were positive for parasites on the blood smear, the geometric mean parasite density was not statistically different between the sites (*P*=0.55; shown in Table 1). Only *Plasmodium falciparum* was detected in the low eBL area. In the high eBL area, most of the parasitaemia-positive cases were infected with *P. falciparum* alone. There were five cases that were infected with both *P. falciparum* and *P. malariae*.

Because we observed significant differences in three parameters (e.g., plasma GPx, EBV viral load and malaria parasitaemia) between the children residing in the low and high eBL risk locations, we wanted to determine if there were correlations between these parameters. We found that plasma GPx levels were significantly lower among participants with the highest EBV viral load (i.e., the highest 20%) and among those with malaria parasites in peripheral blood (shown in Table 3). For children with detectable EBV, log viral loads were also significantly higher in children with positive smears for malaria parasites (3.36 (1.47)) when compared with those without (2.62 (1.00); *P*=0.002). Similar, but nonsignificant, findings were observed in the low and high eBL region as well; mean log EBV viral loads for children with and without malaria parasites residing in the low eBL region were 2.86 (0.87) and 2.49 (0.78), *P*=0.11, whereas mean viral loads among children residing in the high eBL region, with and without malaria parasites, were 3.59 (1.63) and 2.92 (1.38), *P*=0.11.

In a multivariable logistic regression model evaluating residence in the low *vs* high eBL regions and GPx levels while controlling for child age, malaria positivity and log EBV load, higher GPx levels were associated with a decrease (69%) in the likelihood of residing in the high eBL region; the OR (95% CI) for a one-unit increase in GPx (in *μ*g per 100 ml) was 0.31 (0.20–0.49; *P*<0.001). Malaria smear positivity was associated with a seven-fold increase in the likelihood of residing in the high eBL region (OR 7.06; 95% CI: 2.91–17.11; *P*<0.001). Log EBV load was not significantly associated with residence in the high eBL region (*P*=0.99). A similarly fitted model using quartiles of GPx rather than the continuous version indicated that the significant decrease in the likelihood of residing in the high eBL region was observed only for the highest GPx quartile relative to the lowest (data not shown).

## Discussion

The identification of microgeographic variation in eBL incidence in western Kenya (Rainey *et al*, 2007b) led us to ask whether this variation was due to differences in the two known co-factors, EBV and malaria, or whether there were additional environmental factors associated with regions that had higher-than-expected or lower-than-expected cases of eBL. In this study, we performed a cross-sectional survey of children residing in two locations of Nyanza Province that are geographically close but with disparate levels of eBL incidence, and measured EBV viral load and malaria burden in these two groups. We also measured levels of the selenoprotein GPx as well as anthropometric measurements of malnutrition. The results presented here clearly indicate that chronic malnutrition, reduced GPx, increased malaria parasitaemia and increased EBV viral loads are found in children with residence in eBL high-incidence regions when compared with children living in the eBL low-incidence region. This suggests that synergy of multiple risk factors (i.e., malaria and EBV co-infections in conjunction with micronutrient deficiency and chronic malnutrition) may be necessary to set the stage for eBL tumourigenesis.

Malnutrition is a very important indicator for overall child health and is considered a risk factor for infectious and chronic disease susceptibility. In two separate studies in Malawi and Morocco, almost half of children presenting with cancer were malnourished (Israels *et al*, 2008; Tazi *et al*, 2008). Stunting in young children was also shown to impact the development of anti-malaria antibody responses (Fillol *et al*, 2009). These studies combined with ours showing that children from high eBL incidence regions had a higher percentage of stunting relative to areas with no eBL strengthen an argument for chronic malnutrition as a risk factor for eBL.

Plasma GPx has been shown to be a useful biomarker of selenium levels (Jacobson *et al*, 2006). We found significantly reduced levels of plasma GPx levels in children from areas with high eBL incidence that could indicate that there is variability in the micronutrient supply between these two populations and would be consistent with the higher levels of malnutrition that we observed in the high eBL incidence region. However, there was not a direct correlation between children with low GPx levels and children who were stunted, suggesting that the relationship between selenium deficiency and chronic malnutrition is more complex. Low plasma GPx levels could also be an outcome of the high viral load and repeated malaria infections that result in immune activation. Several studies support this possibility. In the first study, respiratory syncytial virus infections result in decline of GPx levels (Hosakote *et al*, 2009). In addition, lower selenium levels were observed in patients with chronic HIV infection and this correlated with immune activation (Stephensen *et al*, 2007). Finally, individuals with *P. vivax* infections were found to have reduced plasma selenium levels compared with healthy subjects (Seyrek *et al*, 2005). Whether repeated *P. falciparum* infections, as occur in malaria holoendemic regions, or the elevated EBV viral loads we observed cause the reduced GPx levels remains to be determined.

The districts where our study took place (Kisumu west and east districts in Nyanza Province, Kenya) were previously combined as one district (Kisumu district), which had been a district classified as having Lake Endemic malaria transmission (Snow *et al*, 1998). We were therefore surprised to find differences in malaria transmission intensity between our two study sites. In the eBL high-incidence region, 62% were parasitaemic whereas it was only 22% in the eBL low-incidence region. However, malaria transmission intensity is based on surveys and rarely are all locations within a district sampled for prevalence of malaria parasitaemia in children. The higher levels of malaria parasitaemia in the high eBL incidence region compared with the low eBL incidence region is consistent with other studies demonstrating a correlation between malaria transmission intensity and eBL risk (Morrow, 1985), but our study is the first to demonstrate this in such a localised area.

We found that the children with the lowest GPx levels had the highest EBV viral loads. In addition, there was a strong trend that found that children with stunting had high EBV viral loads. There is little information on potential links between EBV and micronutrient deficiency or malnutrition. Interestingly, however, is a recent report that shows that the EBV Epstein–Barr nuclear antigen (EBNA)-1 protein can induce ROS that promote genomic instability (Gruhne *et al*, 2009). EBNA-1 is the only EBV latent protein expressed in most cases of eBL and is also expressed in latently infected B cells (Thorley-Lawson and Gross, 2004). We have found that the elevated viral load we observed correlates with increased numbers of latently infected B cells (Wohlford *et al*, unpublished results). Thus, elevated viral loads could result in increased ROS via EBNA-1. In a population with reduced GPx levels, this could be one potential mechanism for an increased risk for eBL. Repeated malaria infections also induce oxidative stress and could potentially enhance the effects of EBNA-1 in B cells.

In this study, we identified reduced plasma levels of the selenoprotein GPx, increased frequency of chronic malnutrition, elevated viral loads and a higher malaria burden in Kenyan children at high risk for eBL compared with those with a lower risk for eBL. Increased GPx levels were associated with a significantly decreased likelihood of residing in the high eBL region after controlling for child age, malaria positivity and log EBV load. A major limitation of our cross-sectional study is that it cannot indicate whether reduced GPx levels is a causal factor for eBL or a marker of oxidative stress resulting from increased infectious burden due to malaria and EBV. Nonetheless, our study highlights the complex relationships between malnutrition and infectious diseases, and is the first to explore whether micronutrients and malnutrition could have a role in eBL aetiology.

## Figures and Tables

**Table 1:** Demographic and clinical characteristics of all study participants by region

**Characteristic**	**eBL low incidence (*n*=107)**	**eBL high incidence (*n*=151)**	***P-*value**
Age (s.d.), years	5.08 (2.57)	4.96 (2.38)	0.70
Gender: *n,* male (%)	51 (48)	68 (45)	0.68
Prevalence+ for parasites^a^: *n* (%)	23 (22)	90 (62)	<0.001
			
*Mean parasite density*^a^ *(s.e.m.): μl*	2262 (616)	7027 (2111)	0.55
Geometric mean	1155	1085	
Mean EBV log viral load^b^ (s.d.)	2.57 (0.81)	3.33 (1.56)	0.001
Mean haemoglobin (s.e.m.): g per 100 ml	12.05 (0.14)	11.50 (0.19)	0.016
Anaemia prevalence^c^: *n* (%)	27 (25)	71 (47)	<0.001

Abbreviations: eBL=endemic Burkitt's lymphoma; EBV=Epstein–Barr virus.

a Malaria parasite assessed was *Plasmodium falciparum*. Mean parasite density among those with parasitaemia.

b Viral load measured in: *n*=127, eBL high; *n*=104, eBL low; means compared among those with detectable levels.

c Anaemia defined as: (Hb <11.0 g per 100 ml 1–4 year olds, or <11.5 g per 100 ml 5–9 year olds).

Groups compared using *t*-tests, Mann–Whitney *U* (parasite density) or Pearson's *χ*^2^ test.

**Table 2:** Demographic and clinical characteristics of study participants by region (*n*=158 with pGPx selenium measurements)

**Characteristic**	**eBL low incidence (*n*=65)**	**eBL high incidence (*n*=93)**	***P*-value**
Age (s.d.), years	4.73 (2.50)	5.15 (2.37)	0.29
Gender: *n*, male (%)	28 (43)	41 (44)	0.90
Prevalence+ for parasites^a^: *n* (%)	12 (19)	59 (63)	<0.0001
			
*Mean parasite density*^a^ *(s.e.m.): μl*	2713 (1122)	8546 (3082)	0.94
Geometric mean	1067	1139	
			
*Mean EBV log viral load*^b^ *(s.d.)*	2.46 (0.88)	3.38 (1.48)	0.001
EBV, detectable virus: *n* (%)	41 (63)	47 (52)	0.16
Mean haemoglobin (s.e.m.): g per 100 ml	11.99 (0.16)	11.17 (0.19)	0.002
Anaemia prevalence^c^: *n* (%)	19 (29)	46 (49)	0.011

Abbreviations: eBL=endemic Burkitt's lymphoma; EBV=Epstein–Barr virus; pGPx=plasma glutathione peroxidase.

a Malaria parasite assessed was *Plasmodium falciparum*. Mean parasite density among those with parasitaemia.

b Viral load measured in: *n*=91, eBL high; *n*=65 in eBL low; means compared among those with detectable levels.

c Anaemia defined as Hb <11.0 g per 100 ml 1–4 year olds, or <11.5 g per 100 ml 5–9 year olds.

Groups compared using *t*-tests, Mann–Whitney *U* (parasite density) or Pearson's *χ*^2^ test.

**Table 3:** Mean pGPx selenium levels (*μ*g per 100 ml) by participant or clinical characteristics

**Characteristic**	***n***	**Mean (s.d.)**	***P*-value** a
*Site*			
Low eBL region	65	3.76 (1.58)	<0.001
High eBL region	93	2.36 (0.65)	
			
*EBV viral load: copies per ml*			
Non-detectable	68	2.92 (1.15)	0.035
2.5–1529	55	3.25 (1.50)	
⩾1530	33	2.50 (1.23)	
			
*Malaria parasites*			
Absent	87	3.19 (1.54)	0.004
Present	71	2.61 (0.89)	
			
*Gender*			
Male	69	3.09 (1.34)	0.19
Female	89	2.81 (1.30)	
			
*Age, years*			
1–4	81	2.97 (1.35)	0.70
5–9	77	2.89 (1.29)	

Abbreviations: eBL=endemic Burkitt's lymphoma; EBV=Epstein–Barr virus; pGPx=plasma glutathione peroxidase.

a Based on the *t*-test or one-way analysis of variance (ANOVA).

**Table 4:** Malnutrition in study participants

**Characteristic**	**eBL low (*n*=104)**	**eBL high (*n*=145)**	***P*-value**	**1–4 years (*n*=128)**	**5–9 years (*n*=121)**	***P-*value**	**Male** **116**	**Female** **133**	***P*-value**
Stunting^a^ *n* (%)	23 (22.1)	58 (40.0)	**0.003**	40 (31.3)	41 (33.9)	0.657	44 (37.9)	37 (27.8)	0.089
Wasting^b^ *n* (%)	21 (23.1)	27 (18.6)	0.467	35 (27.3)	16 (13.2)	**0.006**	22 (19)	29 (21.8)	0.580
Underweight^c^ *n* (%)	32 (30.8)	51 (35.2)	0.390	46 (35.9)	37 (30.6)	0.370	39 (33.6)	44 (33.1)	0.928
Any *n* (%)	46 (44.2)	83 (57.2)	**0.043**	71 (55.5)	58 (47.9)	0.234	63 (54.3)	66 (49.6)	0.460

Abbreviation: eBL=endemic Burkitt's lymphoma.

a Stunting defined as height-for-age *Z*-score <−2.

b Wasting defined as weight-for-height *Z*-score <−2.

c Underweight defined as weight-for-age *Z*-score <−2.

Groups compared using Pearson's *χ*^2^ test.

*Z*-scores were calculated using growth reference curves developed by the National Center for Health Statistics (NCHS) using Epi Info 6.04 (CDC, Atlanta, GA, USA). ‘*n*’ is the number of cases in each category (percent of the total per category). 1. Stunting among the children were significant by geographical location (eBL high or low risk areas (pv<0.003 and there was significant trend by gender (pv=0.089). 2. Any malnutrition among the study children had significant trend by geographical location (pv=0.043). 3. Wasting among the children were significant by age categories (pv=0.006).
